# Understanding the molecular mechanism responsible for developing therapeutic radiation-induced radioresistance of rectal cancer and improving the clinical outcomes of radiotherapy - A review

**DOI:** 10.1080/15384047.2024.2317999

**Published:** 2024-03-06

**Authors:** Samatha M Jain, Shruthi Nagainallur Ravichandran, Makalakshmi Murali Kumar, Antara Banerjee, Alexander Sun-Zhang, Hong Zhang, Rupak Pathak, Xiao-Feng Sun, Surajit Pathak

**Affiliations:** a Faculty of Allied Health Sciences, Chettinad Academy of Research and Education, Chettinad Hospital and Research Institute, Kelambakkam, Chennai, India; b Department of Oncology-Pathology, BioClinicum, Karolinska Institutet, Stockholm, Sweden; c School of Medicine, Department of Medical Sciences, Orebro University, Örebro, Sweden; d Division of Radiation Health, Department of Pharmaceutical Sciences, University of Arkansas for Medical Sciences, Little Rock, AR, USA; e Department of Oncology and Department of Biomedical and Clinical Sciences, Linköping University, Linköping, Sweden

**Keywords:** Rectal cancer, radiotherapy, DNA double-strand breaks, radiosensitizers

## Abstract

Rectal cancer accounts for the second highest cancer-related mortality, which is predominant in Western civilizations. The treatment for rectal cancers includes surgery, radiotherapy, chemotherapy, and immunotherapy. Radiotherapy, specifically external beam radiation therapy, is the most common way to treat rectal cancer because radiation not only limits cancer progression but also significantly reduces the risk of local recurrence. However, therapeutic radiation-induced radioresistance to rectal cancer cells and toxicity to normal tissues are major drawbacks. Therefore, understanding the mechanistic basis of developing radioresistance during and after radiation therapy would provide crucial insight to improve clinical outcomes of radiation therapy for rectal cancer patients. Studies by various groups have shown that radiotherapy-mediated changes in the tumor microenvironment play a crucial role in developing radioresistance. Therapeutic radiation-induced hypoxia and functional alterations in the stromal cells, specifically tumor-associated macrophage (TAM) and cancer-associated fibroblasts (CAF), play a crucial role in developing radioresistance. In addition, signaling pathways, such as – the PI3K/AKT pathway, Wnt/β-catenin signaling, and the hippo pathway, modulate the radiation responsiveness of cancer cells. Different radiosensitizers, such as small molecules, microRNA, nanomaterials, and natural and chemical sensitizers, are being used to increase the effectiveness of radiotherapy. This review highlights the mechanism responsible for developing radioresistance of rectal cancer following radiotherapy and potential strategies to enhance the effectiveness of radiotherapy for better management of rectal cancer.

## GRAPHICAL ABSTRACT

**Figure:**
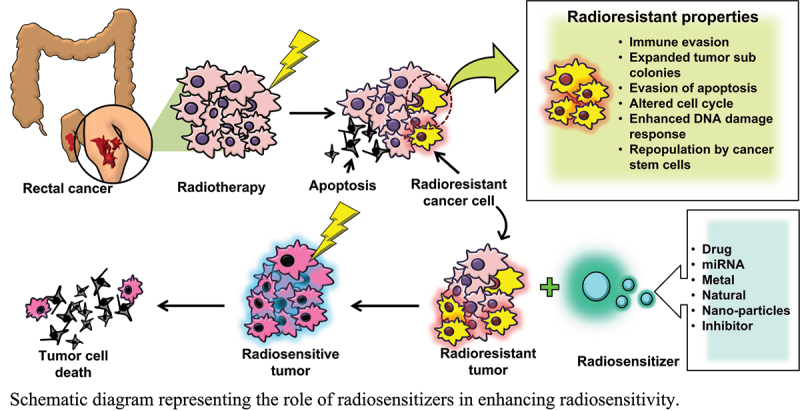

## 1. Radiotherapy in rectal cancer

Rectal cancer (RC) has the second highest incidence of all cancer types and is the second leading cause of cancer-related mortality in Western countries.^1^ In 2020, worldwide, there are estimated to have been approximately 0.7 million new cases of rectal cancer.^2^ More than 90% of rectal cancers are adenocarcinomas, which can be removed by surgical excisions. However, outcomes for surgical excision are dependent on the stage of rectal cancer. It has been found that surgical excision alone, as compared to surgical excision followed by radiotherapy (RT), has a higher risk of local recurrence of rectal cancer.^3^ Preoperative radiotherapy with concurrent chemotherapy followed by surgery significantly reduces the risk of local recurrence.^4^ The current 5-year survival statistics for rectal cancer are most likely due to efforts to reduce loco-regional recurrence rates by better staging, improved surgery, and targeted radiotherapy. A majority of the cases – about two-thirds – occurred in the sigmoid colon or rectum and were identified as Stage II or above, necessitating further treatment with chemotherapy and radiation therapy in addition to the original surgical treatment. Three-dimensional conformal radiation (3DCRT) is the current gold standard for radiotherapy, which allows targeted radiation delivery, dose analysis of target volume and organs at risk (OARs), and dose volume histograms in three dimensions. Because of targeted delivery by 3DCRT, higher dose can specifically be delivered to the tumor, which lowers the risk of developing the symptoms of early and late radiation bowel toxicity. For therapeutic or palliative purposes, 3DCRT has been routinely utilized for medium-risk, locally advanced, and incurable rectal tumors. However, other radiotherapy techniques such as tomotherapy, proton therapy, volumetric arc therapy, and intensity-modulated radiation (IMRT) have been scrutinized to achieve greater conformality index and OAR sparing in treating rectal cancer.^5^ Though RT is recognized as a crucial part of multidisciplinary treatment (MDT) for cancer, certain obstacles are still unaddressed. Development of radiation resistance to rectal cancer (RC) cells, which can adversely affect the treatment outcomes. Although the contribution of different factors in developing radioresistance in RC cells is not fully understood, studies by various groups have shown that intrinsic radioresistance properties, lower apoptosis induction following RT, and functional changes in protooncogenes and tumor-suppressor genes play crucial role in developing RT resistance.^6^ In recent years, significant advancement has been made in radiation delivery techniques and RT optimization. This review aims to summarize the molecular mechanism responsible for therapeutic radioresistance and potential treatment strategies to make RT more efficient and safer for treating patients suffering from RC.

## 2. Mechanisms involved in radioresistance of rectal cancer

Because RC cells gain radioresistance properties over time, RT often fails to completely eradicate RC and thus subsequently increases the risk of local recurrence.^7^ The mechanisms that make RC cells radioresistance are not well understood. Studies by various groups have proposed several mechanisms, which may contribute to the development of radioresistance. The following sections have described some of the mechanisms that may play crucial roles in the development of radioresistance.

### 2.1. Hypoxia

Hypoxia is a characteristic feature of neoplastic cells. A hypoxic microenvironment results from the abnormal vasculature that impairs blood flow, thus impedes the supply of oxygen to the tumor tissue. Inadequate cellular oxygen level enhances radiation resistance by limiting the generation of reactive oxygen species (ROS), which is primarily responsible of cell killing by inflicting various types of DNA damage. The irradiation studies by various groups have shown hypoxia enhances radioresistance.^8^ Hypoxia in the tumor microenvironment can largely be divided into two types: acute or perfusion-limited hypoxia and chronic or persistent hypoxia. Both acute and chronic hypoxia trigger several hypoxia-related tumor responses.^9^ Acute hypoxia results from a temporary occlusion, artery narrowing, and arteriolar vasomotion causing local changes in perfusion and a consequent reduction in oxygen supply.^10^ However, a crucial restriction in oxygen diffusion from tumor microvessels to adjacent tissues results in chronic hypoxia, also known as diffusion-limited hypoxia.^10^ Previously, it was believed that the biological consequences of acute and chronic hypoxia on cells are identical. Recent research has shown that this is not the case. According to recent research, hypoxia-inducible factors (HIF-1) expression in adult organisms promote angiogenesis by transcriptionally activating several angiogenic genes and their receptors, including *VEGF, PlGF, PDGFB, ANGPT1*, and *ANGPT2*. ^11^ By altering Notch signaling, hypoxia can regulate vascular branching. HIF-1α increases the transcriptional activity of the Notch intracellular domain (NICD) by directly binding to it. Furthermore, in the endothelium, HIF-1α and HIF-2α both have transcriptional targets in the Notch ligand Dll4. Following the leading edge, endothelial cells (ECs) start to create tubes that expand the vascular network already in place. In vitro, hypoxia promotes the development of endothelial tubes, and this pro-angiogenic action is reliant on HIF-1α expression in the EC.^12^

### 2.2. Tumour-associated macrophages

**Figure 1.**
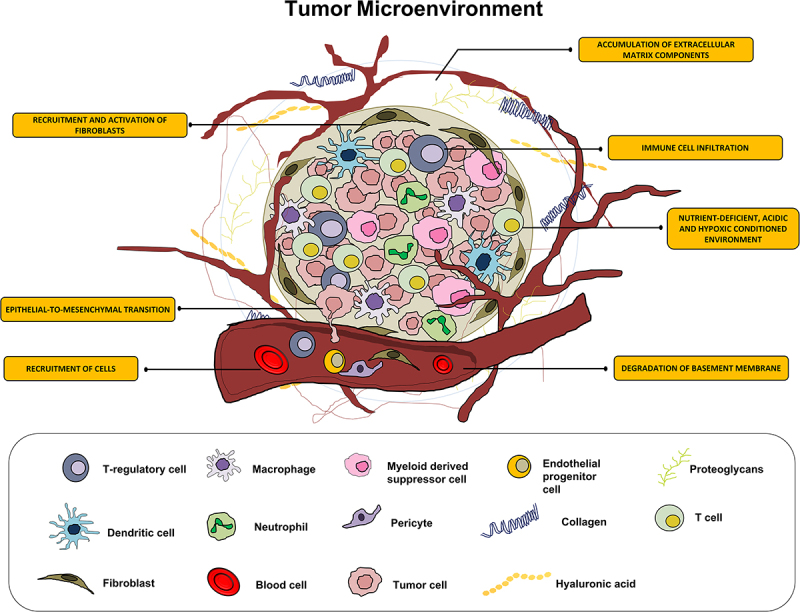
The illustration depicts the contributory role of inflammatory cells in the tumor microenvironment leading to radioresistance.

In RC microenvironment, persistent inflammation results in immune cell infiltration, angiogenesis, and fibroblast proliferation that leads to the development of a distinctive cellular environment (Figure 1 near hear). Among various types of immune cells, tumor-associated macrophages (TAMs) play crucial role in tumor progression and developing radioresistance.^13^ TAMs can exert radioresistance to RC cells with the help of various ways. For example, TAMs, by activating nuclear factor B (NFk-B) signaling synthesizes prostaglandin synthase cyclooxygenase-2 (COX-2), which in turn generates prostaglandin E2 (PGE2), which is known to promote radioresistance.^14,15^ In addition, TAMs generate many antioxidant molecules including manganese superoxide dismutase (MnSOD), which scavenge radiation-induced ROS, and thus provide radioresistance. Finally, TAMs, by activating TNFα signaling suppress radiation-induced cellular damage, thus providing resistance to radiation.^16^

### 2.3. Cancer-associated fibroblasts

There is a strong negative correlation between radiotherapy outcome and the number of fibroblasts in the rectal tumor microenvironment.^17^ Stromal fibroblasts are known to be transdifferentiated into myofibroblasts, also known as cancer-associated fibroblasts (CAFs) when they are recruited into or preexist in the tumor microenvironment by the growth factors like platelet-derived growth factor (PDGF) and transforming growth factor- β (TGF- β) released by cancer cells.^18^ Furthermore, macrophage-derived TNF-α promotes fibroblast growth.^19^ CAFs cause tumor radioresistance. Studies in rectal cancer have shown in the presence of M2 macrophages, carcinoembryonic antigen (CEA) can induce radioresistance to the rectal tumors.^20^ Further studies revealed that exosomes in the conditioned medium (CM) of CAFs are responsible factors for such phenotypes. Exosomes derived from CAF’s CM also increase the colorectal cancer stem cell-like phenotypes in tumor and decrease the radiosensitivity.^21^ For instance, CAFs release fibroblast growth factor 4 (FGF4), insulin-like growth factor 2 (IGF2), and epidermal growth factor (EGF), all of which promote the proliferation and survival of cancer cells after radiotherapy.^22,23^ Moreover, IGF1/2, C-X-C motif chemokine ligand 12 (CXCL12; also known as stromal cell-derived factor 1 or SDF-1), and β-hydroxybutyrate produced by CAFs have been shown to promote autophagy in irradiated cancer cells and hasten the recovery and regeneration of tumors.^24^

## 3. Pathways that contribute to rectal cancer radioresistance

**Figure 2.**
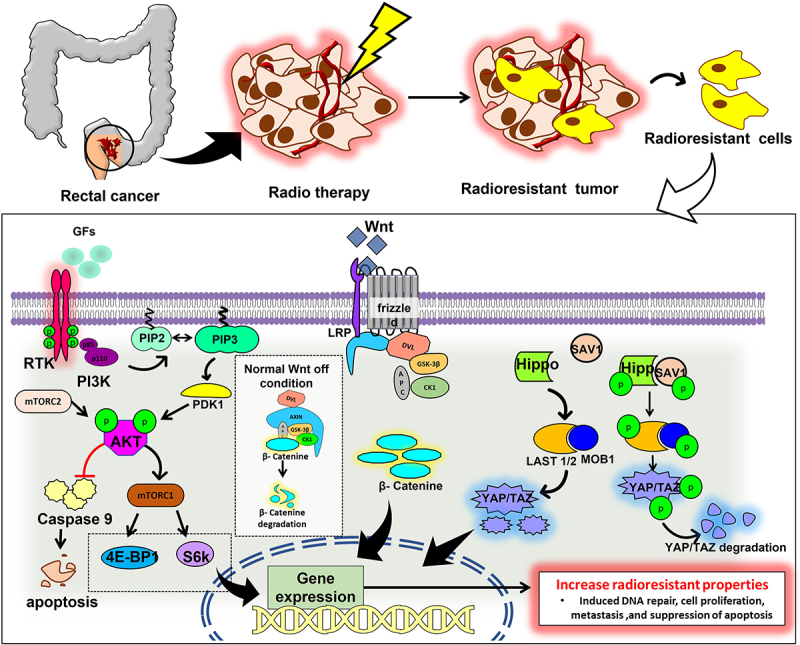
The diagram represents pathways associated with rectal cancer radioresistance.

Even though RT has considerably improved cancer patients general health and well-being, its efficacy has still been severely constrained by intrinsic or acquired tumor radioresistance. Radiation kills cancer cells primarily by damaging their DNA. It simultaneously engages several signaling pathways, including those regulated by AKT/PI3K/mTOR pathway, Wnt/β-catenin pathway, Yap/Taz pathway, Hedgehog and NF-κB (Figure 2). Additionally, evidence supports the role of signaling pathways in enhancing cancer cells’ intrinsic radioresistance, which is achieved by activating several vital genes necessary for DNA repair, cell survival, proliferation, and cancer stem cell (CSC) stemness.^25^ Many different pathways involved in RT resistance cancer development have been extensively studied. The most clinically relevant among these pathways are discussed below.

### 3.1. PI3K/Akt/mTOR signalling pathway

The phosphatidylinositol-3-kinase/Akt/mammalian target of rapamycin (PI3K/Akt/mTOR) pathway promotes cell growth and proliferation, is frequently dysregulated in rectal cancer due to deletion, mutation, methylation, amplification, and post-translational changes. This signaling pathway also plays a role in malignant transformation, apoptosis, metastasis, tumor growth, and radioresistance.^26^ Studies by various groups have shown that overexpression of Potassium Voltage-Gated Channel Subfamily E Regulatory Subunit 4 (KCNE4) activates PI3K/AKT signaling pathway.^27^ miRNAs regulate the PI3K/AKT pathway’s activity as upstream regulators and downstream effectors. They also regulate the development, spread, and metastasis of cancer. Furthermore, the abnormal expressions of miRNAs promote radioresistance. Through the inhibition of CLCA4 and the activation of the PI3K/Akt signaling pathway, it has been found that miR-590-3p enhances the resistance of RC cells to RT.^28^ Moreover, the mTOR signaling pathway promotes the epithelial-to-mesenchymal transition (EMT) in tumor tissues essential for the CSC homing and induction of radioresistance.^29^ Additionally, the PI3K pathway activation promotes cancer stem cell proliferation, migration, and stemness. Experimental data suggest that the number of CSC can vary between tumors, even those with the same histopathological type and that a higher percentage of cancer stem cells are associated with higher radioresistance. In high nutrient-rich conditions, Akt regulates ROS production, which can regulate the level of radioresistance in cancer cells.^30,31^ Activated Akt1 can promote the repair of IR-induced DNA-DSB, which increases post-irradiation cell survival.^32^ By regulating E2F1 and causing a decrease in CD151 through the inhibition of AKT/mTOR/cyclinD1 signaling, also RC stem cells’ RT resistance was decreased by reduction of the lncRNA DLGAP1-AS2, demonstrating that one efficient way to improve the radiosensitivity of RC stem cells is through lncRNA DLGAP1-AS2 suppression.^33^

### 3.2. Wnt/β-catenin signalling pathway

The Wnt/β-catenin signaling system is extremely conserved, with important regulatory functions in cell stem cell development, differentiation, proliferation, migration, and apoptosis. Wnts, as significant messengers in the RC microenvironment, primarily contribute to cancer development through canonical Wnt signaling. Wnt exerts radiation resistance by promoting DNA damage repair and preventing apoptosis.^34^ It has been shown that Ionizing Radiation can cause stromal cells and cancer cells to secrete Wnt. IR-mediated Wnt overexpression is one of the key factors for acquired radioresistance. For instance, IR-induced Wnt16B production by fibroblasts encourages cancer cells to undergo EMT, which increases their resistance to IR.^35^ Wnts are also engaged in stromal cell communication. For instance, IR-induced overexpression of Wnt16B in fibroblasts activates the canonical Wnt signaling pathway and controls the development of regulatory T cells, leading to cancer immune evasion.^36^ IR prompts cancer cells and stromal cells to produce Wnts and various other coactivators, which work together to shield cancer cells from radiation-induced damage. It has been shown that the Wnt signaling pathway is involved in controlling oxidative stress since the expression of the hydrogen peroxide detoxifying enzyme catalase was lowered and ROS levels were elevated in cells after IR treatment when β-catenin was absent.^37^ By balancing the Wnt-TCF signaling route, which is mostly proliferative, and the forkhead box O (FOXO) signaling pathway, β -catenin can guard against oxidative stress (mainly stress response). By regulating the homeostasis between the forkhead box O (FOXO) signaling pathway and the TCF signaling pathway, β-catenin can also guard against oxidative stress (mainly stress response).^38^ β-catenin directly targets the DNA ligase LIG4, which is involved in DNA double-strand break repair. Trans activation of LIG4 induces the non-homologous end-joining repair in rectal cancer. Therefore, LIG4 inhibition can increase the radiosensitivity of the RC. Extreme radiosensitization is seen in LIG4 somatic knockout cell lines, indicating that LIG4 is necessary for DSB repair.^39^ The expression of E3 ubiquitin-protein ligase, RNF6, in radioresistant RC is higher which promotes the activation of Wnt signaling. The over-expression of RNF6 increases the resistance to radiotherapy in rectal cancer cells.^40^

### 3.3. Hippo signalling pathway

Hippo signaling is a ubiquitous evolutionary route that regulates stem cell self-renewal, tissue regeneration, cell proliferation, and organ growth.^41^ Through the activation of Hippo signaling and the upregulation of TAZ and CDK5 contributes to the development of cancer and RT resistance.^42^ The carcinogenic function of YAP1, a transcriptional coactivator and the primary target of the Hippo pathway, has been extensively studied.^43^ The overexpression of YAP1 in RC samples was confirmed by Yao et al.. (2022),^44^ and the authors also demonstrated YAP1 binds to ANKHD1. The physical interaction between ANKHD1 and YAP1 in this study suggests that, rather than activating the Hippo pathway, ANKHD1 directly influences YAP1 transcriptional activity. Hence, ANKHD1 expression may impact RC cells’ radiosensitivity. Therefore, ANKHD1/MALAT1/YAP1 was suggested as a potential interactive regulatory loop that might synergistically control the transcriptional activity of YAP1 and activate AKT, which in turn affects IR-induced DNA damage repair in RC. Collectively, they propose a possible specific mechanism for the YAP1/AKT axis downstream of the ANKHD1/MALAT1/YAP1 loop, which may be a therapeutic target for treating RC.

A positive correlation exists between YAP1 activation and inherent radioresistance of many cancer, while the precise mechanisms underlying this YAP1 effect are still unclear, a previous study suggests that it generally involves the YAP1 function in the transactivation of gene expressions necessary for DNA repair (p73, etc.), cellular proliferation (EGFR/HER, Axl, cell cycle genes, MAPK, etc.), cell survival (survivin, Bcl-2/Bcl-XL, etc.) and cancer cell stemness (SOX2, CTGF, Cyr61, etc.). Therefore, YAP1 activates prosurvival pathways, by aiding DNA repair, blocking apoptosis, and maintaining cancer cell stemness; in addition, YAP1 can decrease radiation-induced cytotoxicity and increase the radioresistance of cancer cells.^45^

## 4. Factors contributing to radiation resistance and local recurrence of rectal cancer following radiation therapy

The extent of tumor regression, the response rate, and the response persistence are all responsible for radiosensitivity. Numerous factors such as the potential to restore damage, hypoxia, cell cycle stage, and growth factors modulate radiosensitivity. Additionally, it has been shown that the size of the initial tumor determines the outcome of radiation therapy, specifically the local recurrence. Factors that contribute to local recurrence include the exclusion of a part of tumor from the radiation field, uniform dose distribution leaving some cancer cells unaffected, and cancer metastasis. The inability to complete the elimination of tumor cells following primary RT is considered to be the main factor for local tumor recurrence. Hypoxia plays a crucial role in preventing the complete elimination of cancer cells following RT. Hypoxia provides resistance to radiation by several different processes. Therefore, reducing hypoxia in the tumor microenvironment is a potent strategy for tumor cell radiosensitization. Hypoxia in the tumor microenvironment may develop because of disrupted angiogenesis and desmoplasia (i.e., excessive connective tissue formation). Under normoxic conditions, ionizing radiation destroys mitochondria, which increases the generation of reactive oxygen species, which damages DNA and triggers cell death. However, in hypoxic tumors, RT results in less DNA damage.^46^ Mechanistic studies revealed that hypoxia-induced stabilization of hypoxia-inducible factor 1 (HIF1) leads to the buildup of NADPH and the induction of glycolysis, which in turn scavenges ROS, thus provides radioresistance.^47^

Additionally, glycolysis causes lactate to build up, which upregulates HIF1 to end the cycle of hypoxia-mediated RT resistance. Since hypoxic cells sustain less DNA damage than normoxic cells following radiation exposure, the hypoxic cells exhibit higher radioresistance compared to normoxic cells. Lesser bioavailability of molecular oxygen causes less ROS generation following irradiation. ROS plays a crucial role in cell death by inducing DNA damage. Most of the DNA damage caused by free radicals can be chemically repaired in the absence of molecular oxygen, thus providing radioresistance. Consequently, fewer DNA double-strand breaks (DSBs) are created when hypoxic tumor cells are exposed to radiation, as shown by a decrease in the amount of H2AX foci (a marker for DSBs) in hypoxic regions. HIFs control how cells react to low oxygen levels.^48,49^ Cellular adaptations to hypoxia are driven by the HIF-transcriptional program. The activity of CDKs is crucial for the advancement of the cell cycle from the G1 to the S phase. The E2F transcription factors are released after the pocket proteins (pRB, p107, and p130) are phosphorylated and inactive, starting the transcriptional program linked to the S-phase entrance. Cyclin D2 expression and the CDK inhibitors p21 and p27 are induced by hypoxia through HIF-dependent regulation of c-Myc, which results in cell cycle arrest.^50–52^ Additionally, the minichromosome maintenance (MCM) proteins, essential for the success of DNA replication, can directly interact with HIF1a and reduce their activity.^53^ These findings demonstrate that hypoxia directly influences cell cycle progression during the G1/S phase transition through the activity of HIFs. Cancer-associated fibroblasts are frequently the predominant cell type in the TME. CAFs were once thought to have little significance in the TME but are now understood to play a crucial role in cancer development. It has recently been clear that there are several subsets of CAFs, some of which exhibit protumorigenic features while others exhibit stronger antitumorigenic traits. Adipocytes, endothelial cells, stellate cells, resident fibroblasts, bone marrow-derived mesenchymal cells, and mesenchymal stem cells are only a few of the cell types that can give rise to CAFs.^52^ This can vary not just between tumor types but also between tumor beginning and progression stages.^53,54^ The intratumoural CAF population in various solid tumors comprises many subsets that can react differentially to different stromal stimuli, exhibit unique secretory phenotypes, and carry out specific biological activities in the TME.^55,56^

## 5. Mechanisms of sensitizing tumors to radiation

### 5.1. Increased production of reactive oxygen species

ROS plays myriad functions in cancer pathogenesis. ROS acts as mitogens at higher level, triggering cancer cell proliferation. ROS-induced DNA mutations can inactivate tumor-suppressor genes to promote carcinogenesis. ROS produced by healthy cells is efficiently eliminated by cellular antioxidant systems. However, excessive ROS produced by cancer cells may cause various types of DNA damage, which can contribute to cancer pathogenesis. Among other mutagenic changes, DNA oxidation can result in changes to DNA bases or double helix breakage. Direct strand excision and oxidative damage to the pyrimidine and purine bases are two effects of hydroxyl radical stress on DNA. The radical-induced abstraction of a proton from any location of the deoxyribose initiates this process, which can produce a variety of compounds. Through oxidizing nucleoside bases (such as guanine, which results in the creation of 8-oxo guanine), ROS have also been found to directly cause various types of DNA damage. A comparison of the biological properties of citrus pectin and apple pectin reveals that both can cause oxidative DNA damage that can result in G-T or G-A transversions if left unrepaired. When they occur concurrently on opposing strands, the BER pathway normally detects and repairs oxidized bases, but when this happens, it can result in the formation of DSBs.^57^ In addition, an increase in ROS causes mitochondrial DNA strand breakage, lesions, and degradation.^58^ By altering mitochondrial permeability, which is related to p53-dependent apoptosis (PUMA) can increase the generation of ROS. The neutrophil cytosol factor 2 gene codes for p67phox, a subunit of the NADPH oxidase complex that may be crucial in the rise in cytosolic O2− levels. These interplays suggest that elevated ROS buildup stabilizes p53 protein and makes cells susceptible to activation of apoptosis. Inducing tumor cell death in a p53-dependent manner and elevating intracellular ROS levels are two significant outcomes of radiation exposure.^59^

**Figure 3.**
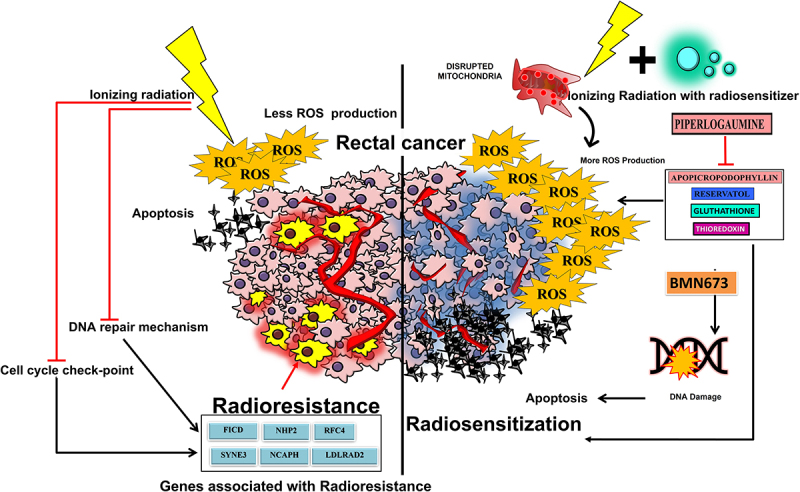
The figure depicts the wide play of ROS and its molecular mechanism in rectal cancer.

Pro-drugs that specifically raise the level of reactive oxygen species in cancer cells while not affecting normal cells may function as radiosensitizers for radiation therapy with no adverse effects.^54,55^ (Figure 3). ROS can harm cancerous cells in various ways, including by causing DNA damage and altering gene transcription. Hydrogen peroxide (H_2_O_2_), reactive hydroxyl radicals (−OH), and anion superoxide make up much of the ROS generated (O_2_-). There is unmistakable preclinical and clinical evidence that the intensity, kind, and duration of ROS exposure all affect how much harm is caused by them. Elevated levels of ROS can cause cell cycle rearrangement and cellular death after being induced.^57^ Therefore, the formation of ROS may be increased if radiosensitizers are present in the cancer cells. The induction of apoptosis, which was noted in a few studies, may be responsible for these effects.^60,61^ Early investigations found that reducing GSH could increase the radiosensitivity of cell lines for squamous cell cancer that are radiosensitive.^62^ Recently published research has outlined Nrf2‘s function in radioresistance. The interaction of the repressor protein Keap1 with Nrf2 results in its normal degradation. Lack of Keap1-Nrf2 relationship and Keap1 loss-of-function mutations result in abnormal Nrf2 activation and radiation resistance. The control of antioxidants by the cooperative actions of thioredoxin and GSH is one of the additional mechanisms that impart radioresistance^63^ stem cells from cancer. Active ROS-scavenging mechanisms in cancer stem cells result in reduced ROS levels, less radiation-induced DNA damage, and higher radioresistance.^64^

### 5.2. DNA damage

Double strand breaks (DSBs) are the most toxic damage among all forms of DNA damage induced by ionizing radiation. The extent of unrepaired DSBs is the primary determinant of cellular death following radiation exposure, while misrepaired DSB can lead to carcinogenesis.^65^ Therefore, suppressing or inhibiting DSB repair following radiation exposure is one of the most popular strategies for radiosensitization. These lesions can cause several cellular DNA damage responses (DDRs) such as cell cycle arrest, DNA repair, and activation of DNA damage capable of detecting early transduction pathways. These insulating DDRs undoubtedly confer tumor radioresistance. It has become popular to target DDR signaling pathways to overcome tumor radioresistance, and significant developments and discoveries have already been made in this area in the past decade. The degree of radiation-induced DNA damage varies depending on the number of ionization processes and how close they are to the double helix, as well as on the ability of cells to remove free radicals and the effectiveness of DNA damage repair pathways. To prevent or overcome PARPi resistance and improve the tumor response to therapy, it may be possible to combine PARPi with other chemotherapeutics or RT. Combining PARPi with RT is desirable for increasing PARPi’s effectiveness.^66–70^ Investigations have suggested that several clinically significant PARPi may be radiosensitizable; however, most of these studies, except BMN673, only demonstrate modest radiosensitization. While not affecting normal cells, BMN673 showed strong radiosensitization in cancer cells.^71–79^ The “optimal” clinical radiosensitizer is BMN673, which has different DSB repair modulatory actions and appears to offer unexpectedly adaptable drug therapy regimes.^80^

There is a growing body of evidence that BMN673 radiosensitizes cancer cells by compromising their DNA double-strand break (DSB) repair pathways. One of the current hypotheses is that BMN673 enhances error-prone DSB processing that robustly enhances cell death by strongly inhibiting classical non-homologous end-joining (c-NHEJ) and dramatically increasing DSB end-resection reciprocally.

IR results in DSBs and triggers the quick synthesis of H2AX, a protein that indicates a cell’s inability to repair DSBs. Using immunofluorescence; scientists assessed H2AX in XRCC2-deficient RC cells to determine how olaparib influenced IR-induced DNA damage. After 48 hours, olaparib treatment alone produced no discernible DNA damage, whereas olaparib treatment in conjunction with IR led to greater levels of H2AX. Importantly, irradiated XRCC2-deficient cells had more H2AX foci than XRCC2-expressing cells 48 hours after receiving olaparib. These results showed that olaparib+IR therapy increased DNA damage higher in XRCC2-deficient RC cells than in empty vector control cells. The HRR activity of tumor cells is a significant factor in treatment outcomes with a PARP1 inhibitor. The XRCC2 protein interacts in HRR through various mechanisms, including the DSB repair process. It is believed that the inhibition of base excision repair induces radiosensitization driven on by treatment with a PARP1 inhibitor. SSBs eventually turn into 1-ended DSBs when they collide with replication forks because of the delay in their repair. These DSBs can only be repaired by HRR.^81^ Transmembrane molecule Bcl-xL, which is highly expressed in colorectal cancer tissues, may cause the disease to become resistant to treatment. Bcl-xL controls the permeability of the mitochondrial membrane, prevents the cytochrome c release, and therefore inhibits apoptosis. Chemotherapy or radiotherapy may have a more significant pro-apoptotic effect if it uses therapeutic agents that target Bcl-xL by DNAzymes. SW480 and SW837 cells were subjected to these Bcl-xL DNAzymes, which successfully decreased Bcl-xL expression and induced cell apoptosis. Therefore, these Bcl-xL targeting enzymes (DT882, DT883, and DT884) suppressed the expression of Bcl-xL and made RC cells more sensitive to 5-FU and radiotherapy.^82,83^

Regardless of the tumor genotype, oxidative stress is a prominent characteristic of CRC. The upregulation of NAD(P)H: quinone oxidoreductase 1 (NQO1) in neoplastic colon tissue is one of the factors driving this process. As a cytosolic flavoprotein antioxidant enzyme that uses NADH/NADPH to catalyze quinone reduction, NQO1 protects cells from oxidative stress by halting the generation of reactive oxygen species. Therefore, targeting NQO1 may be a sensible strategy to promote cell death and maximize the curative effect of radiotherapy, which is frequently used to treat rectal cancer. In this study, it is speculated that napabucasin’s antiangiogenic effects could normalize vascularization in CRC tumor models, improving oxygenation and boosting ROS production and ionizing radiation. The DNA damage response is activated when there are higher concentrations of ROS, such as H_2_O_2_, which causes ATM to be autophosphorylated and produce pATM. γH2AX expression is upregulated as another sign of oxidative stress and the initiation of the DNA damage pathway.^84^

It is also intriguing to note that persistently elevated ROS promotes H2AX protein degradation, which is connected to lowered levels of H2AX and, consequently, improved platinum sensitivity in triple-negative breast cancer. On the other hand, acute oxidative stress greatly enhances DDR signaling and H2AX activation. This has been linked to worse outcomes for colorectal, breast, and lung cancer and has been mentioned to blunt the therapeutic response to radiation and chemotherapy. Additionally, ROS accumulation affects cell fate differently depending on the p53 status, with more apoptosis occurring in cells with functional WT p53. Depending on the level of ROS, p53 plays a critical role in controlling pro- and antioxidant-gene expression. p53 turns on antioxidant genes when ROS levels are low and pro-oxidant genes when ROS levels are high. In addition to these other mechanisms, thioredoxin, and GSH work together to regulate antioxidants, which confers radioresistance. Since cancer stem cells have active ROS-scavenging mechanisms, they exhibit lower ROS levels, less radiation-induced DNA damage, and higher radioresistance. The ROS function in response to DNA damage is complex and pleomorphic.^63,85^ Therefore, it is essential to distinguish between the role of ROS in regulating DDR components and the impact of oxidative stress that result in DNA damage and downstream initiation of DDR (signaling and effectors). There is significant proof that, in a context-specific fashion, ROS dysregulation plays a role in the development of cancer as well as chemoresistance and radioresistance.

## 6. Radiosensitizers as therapeutic strategy in rectal cancer

It is critically necessary to develop extremely effective radiosensitizers to combat the cancer cells’ ionizing radiation resistance, which is the primary cause of radiotherapy’s failure.

### 6.1. Small molecular drugs as radiosensitizers

Due to the suppression of mitochondrial complex I and activation of the AMPK signaling system, the anti-diabetic medications metformin and phenformin effectively eliminate cancer cells and reduce metastases.^86^ Together, the findings of this study reveal that metformin and phenformin inhibit mitochondrial complex I, which prevents RC cells from aerobic respiration and increases radioresponse. Considering human clinical trials commencing in association with radiotherapy, the radiosensitizing impact of metformin has been recognized, but for phenformin, the clinical landscape is yet entirely unexplored.^87^

Currently, the topoisomerase I inhibitor irinotecan, an analogue of camptothecin, is used to treat metastatic colorectal cancer. It has been discovered that camptothecin derivatives radiosensitize many cancer cell types. According to the results of this study, irinotecan causes radiosensitization in HT29 and SW620 cells, and the SER increases as drug concentrations increase. This impact in p53-mutant colorectal cancer cells is attributed to the activation of the DNA damage response system, which results in a considerable G2/M phase arrest and accelerated apoptosis. This mechanism most likely happens through the ATM/Chk/Cdc25C/Cdc2 pathway. Large-scale clinical trials are necessary to examine irinotecan’s effectiveness and radiosensitizing effects.^88^ The receptor tyrosine kinase C-Met regulates several cancer-related activities. The downstream signal transduction pathways, including ERK and AKT, were modified by the c-Met inhibitor crizotinib, which also successfully prevented radiation-induced c-Met activation. This study reveals how the c-Met inhibitor crizotinib can radiosensitize colorectal cancer with the KRAS mutation. According to our tissue microarray investigation, colorectal carcinoma with the KRAS mutant protein expresses c-Met highly. Radiosensitize cancer resistant to EGFR-targeted therapy by targeting this kinase.^89^ Studies on Dactolisib (BEZ235), a dual inhibitor PI3K/mTOR, have shown that BEZ235 increases radiosensitivity in RC cell lines and therefore decreases cell survival in RC cell lines. The degradation of DNA repair mechanisms and the lowering of radiation-induced AKT/mTOR signaling activation are two potential mechanisms for radiosensitizing effects. According to a study, pre-treatment with BEZ235 and radiation could be an efficient neoadjuvant therapy for rectal cancer, including tumors with the K-RAS mutation.^90^ Thymidine phosphorylase inhibitor tipiracil hydrochloride (TPI) and trifluridine (FTD) combine in a unique oral formulation known as FTD/TPI. Although there is currently no reliable molecular biomarker that could be used to identify patients affected with colorectal cancer who might most benefit from FTD/TPI-based chemoradiation therapy, the outcome of this analysis suggests that FTD/TPI treatment has a strong radiosensitizing effect at the cellular level and that its effectiveness is comparable to that of capecitabine when combined with radiation. In an ongoing clinical trial (NCT04177602), the preclinical experimental result demonstrates radio chemotherapy with FTD/TPI for advanced rectal cancer would be evaluated.^91^ A possible anticancer drug has been discovered in the form of β-apopicropodophyllin (APP), a derivative of podophyllotoxin (PPT). Conventional radiation therapy typically has a therapeutic effect by the indirect action of free radicals created by the radiolysis of water, followed by the oxidation of biomolecules. Small compounds that increase free radical generation can intensify these effects. These results suggest that APP is a good option for radiosensitizers that promote apoptosis through DNA damage and the generation of free radicals like ROS.^92^ Together, the findings of this and previous studies indicate that JNC-1043 promotes apoptosis, which is mediated by mitochondrial ROS, and has anticancer effects against RC cells.^93^

Resistance to chemotherapy drugs is also another main problem in the effective treatment of several types of cancers. Anthracycline antibiotic doxorubicin (DOX) is a chemotherapeutic agent that is most frequently used to treat cancer, either as a singular treatment or in combination with other chemotherapeutic drugs or radiation. Gold nanoparticles (GNPs) have been applied effectively as radiation-sensitizing agents. GNPs have been applied effectively as radiation-sensitizing agents. Studies indicate that understanding the molecular processes and interactions between GNP-F and DOX as radiosensitizer recreational drugs might help to increase the efficacy of RC treatment.^94^ In RC cells, the combination of temsirolimus and chloroquine demonstrated radiosensitizing effects. Patients receiving neoadjuvant CRT for rectal cancer may benefit from this combination therapy, which combines chemotherapy and RT.^95^

### 6.2. miRNA as a radiosensitizer

Numerous cancer-related processes, including cell cycle, invasion, proliferation, migration, apoptosis, radiosensitization, and treatment resistance, have been linked to the emergence and distribution of miRNAs. miR-32-5p is overexpressed in colorectal cancer tissues and is significantly connected using clinically poor prognosis and clinicopathological characteristics. Furthermore, it was shown that miR-32-5p negatively controlled the transducer of ERBB2, 1(TOB1) expression to regulate radiosensitivity and prevent colorectal cancer cells’ invasion and metastasis. Findings reveal the function of miR-32-5p upregulated in colorectal cancer tissues and positively correlated with clinicopathological features and survival. Mechanistically, the knockdown of miR-32-5p enhanced the radiosensitization and suppressed metastasis of colorectal cancer through directly binding to the 3ʹ-UTR of TOB1 mRNA Taken together, miR-32-5p acts as an oncogenic factor in the development of colorectal cancer, which may offer a novel as well as a useful biomarker for the detection of colorectal cancer for diagnosis and prognosis and a potentially successful target for treatment.^96^

### 6.3. Inhibitory molecules as radiosensitizers

The SCF (Skp1, Cullins, F-box proteins) multi-subunit E3 ubiquitin ligase, also known as CRL (Cullin-RING ubiquitin Ligase) is the largest E3 ubiquitin ligase family, which regulates different biological process including cell cycle and replication, by promoting ubiquitination of various regulator proteins. The cullin family of proteins has been well characterized as the major substrates for neddylation, a post-translation modification process that adds ubiquitin-like protein NEDD8 (neural precursor cell-expressed developmentally downregulated protein 8) to target proteins. The activity of SCF E3 ubiquitin ligases requires cullin neddylation. Neddylation is mediated by NEDD8 activating enzyme E1 (NAE). Another study revealed the radiosensitizing activity of MLN4924, a small molecule inhibitor of SCF E3, enhanced radiation-induced G2/M arrest, apoptosis, and DNA damage response and also elucidated its mechanisms of action, which include accumulation of p27 therefore, provides an appealing piece of evidence for future development of MLN4924 as a novel radiosensitizing agent against colorectal cancer.^97^

### 6.4. Nano-based radiosensitizers

Because of their higher bioavailability, the use of nanoparticles to develop radiosensitizer has gained significant interest. Additionally, nanoparticles have the tumor-specific enhanced permeability and retention (EPR) properties.^98^ The great majority of these nanosystems can increase tumor-anti-cancer drug enrichment and reduce *in vivo* systemic toxicity, considerably improving the efficacy of cancer treatment. Curcumin-FFE-CS-EE, a drug-peptide conjugate, can self-assemble into small-molecule hydrogels and nanofibers under mild conditions, attaining controls of self-assembled nanostructure and fixed drug loading content as high as 38.2%. A novel curcumin-based supramolecular nanofiber that performs admirably in increasing cancer cell tumor radiosensitivity to IR attributable to a self-assembling peptide. Because of the supramolecular nanostructure, Cur-SNFs can function as a much superior radiosensitizer to sensitize rectal cancer cells to IR than free curcumin, according to in vitro and in vivo radiosensitization experiments.^99^ PEGylated GQD-decorated silver Nano prisms (pGAgNPs) showed improved intracellular absorption when both types of nanoparticles were evaluated *in* *vitro* in radiation-sensitive rectal cancer cells (pAgNPs). pGAgNPs and pAgNPs may radiosensitize tumors and boost the effectiveness of radiation therapy without needing to increase radiation doses, according to *in* *vitro* and *in* *vivo* evidence.^100^ According to research, Fe3O4@Cus-PEG NPs are a promising nano radio-sensitizing agent, and the presence of nanoparticles enhanced the effects of X-ray radiation.^101^

### 6.5. Natural sensitizers

Long pepper naturally produces piperlongumine (PL), which is known to kill tumor cells by increasing cellular ROS generation. Excessive ROS generation inhibits glutathione and thioredoxin, which are known cellular antioxidants. PL has been shown to enhance the radiosensitivity of RC cells in aerobic and hypoxic conditions. This is achieved by cell cycle arrest, ROS-mediated DNA damage, and suppressing cellular respiration. Moreover, by inhibiting both the GSH and Trx systems, PL dramatically increased the radioresponse of colorectal tumors. The findings justify using PL as a radiosensitizer for colorectal cancer and urge for more research.^85^

Allicin, also known as diallyl thiosulfate, is a versatile chemical molecule containing sulfur and a key lipid-soluble active component isolated from garlic (*Allium sativum L*.). Allicin is employed as a potential sensitizer for tumor irradiation in clinical practice since it increases the sensitivity of X-ray radiation treatment for RC. In addition, its mechanism is related to suppressing the NF-κB signaling pathway. These findings suggest that allicin can be used as a potential sensitizer for tumor RT.^102^

### 6.6. Metal-based sensitizers

In contrast to platinum-based medications, a transition metal from the platinum family is ruthenium (Ru). It was determined whether the chiral Ru (II)-arene complexes AH54 and AH63 affected human colorectal cancer cells biologically. Recently demonstrated clinically relevant RT dosages significantly increase radiosensitization, an apparent biological mode of action involving DNA damage generated by these substances, and higher radiosensitizing activity in p53-wild type cells compared to p53-null or p53-mutated cells.^103^ It was suggested that these compounds should be further researched as anticancer medicines in conjunction with RT if these results are validated in *in-vivo* preclinical models.^104^

### 6.7. Chemical sensitizer

About 10% of the glucose that is produced during glycolysis is diverted to the *de novo* serine synthesis pathway (SSP) by the first and rate-limiting enzyme, phosphoglycerate dehydrogenase (PHGDH).^105^ The results showed that while the inherent radiosensitivity is unaffected, modulating the de novo serine synthesis pathway by inhibiting PHGDH improves the radiation response of hypoxic colorectal cancer cells. This PHGDH inhibition-mediated radio-sensitization, at least in part, is the result of dysfunctional mitochondrial metabolism and ROS imbalance.^106^ More investigation into the extracellular serine dependency of cancer cells and the development of therapeutic drugs that target PHGDH will undoubtedly lead to exciting potential in cancer research (Table 1).^106^

**Table 1.** List of currently available radiosensitizers in rectal cancer therapy.

Radiosensitizers	Pathways/targets	Types of cancers	Modes of action	References
Irinotecan	ATM/Chk/Cdc25C/Cdc2	Colorectal	Topoisomerase -I inhibitor	^87^
Crizotinib	ERK/AKT	Colorectal	C-met inhibitor	^88^
Dactolisib	PI3K/AKT	Rectal	PI3K/AKT inhibitors	^89^
miR-32-5P	TOB1	Colorectal	TOB1 -ve regulation	^96^
MLN4924	P27	Colorectal	SCF E3 ligase inhibitor	^97^
CUR-SNFs	NFk-B	Colorectal	Increase the efficacy of radiation	^98^
Piperlongumine	INF-α, NFk-B, ERK, AKT	Rectal	Inhibits the glutathione and thioredoxin systems	^85^
Alicin	NFk-B	Colorectal	Increase X-ray sensitivity	^102^
Ruthenium	P53	Colorerectal	DNA damage	^104^
PHGDH	Glycolysis	Colorectal	Dysfunctional mitochondrial metabolism and a disturbed ROS balance	^85,106^
JP-1201	XIAP, PRAP-1	Colorectal	Double-strand DNA break	^107^
Ganetespib	HSP90	Colorectal	Promote apoptosis	^108^
Selinexor	XOP1	Rectal Cancer	Increased apoptosis and decreased proliferation	^109^
c-phycocyanin	COX-2	Colon cancer	Anti-inflammatory effect	^110^
miR-130a 4	SOX	Rectal	Disrupts EMT and induce DNA damage	^111^

## 7. Conclusion and future perspectives

RC is the third most frequently diagnosed cancer and the third leading cause of cancer death.^2^ Treatment strategies include chemotherapy, surgery, immunotherapy and RT. RT, primarily by inducing DNA damage, eradicates cancer cells, thus reducing tumor size. Although RT has made a significant advancement, tumor-specific radiation delivery without damaging the surrounding healthy tissue is still an unmet goal. Tumor radiosensitivity can further be enhanced by treating patients with chemicals/pharmacological substances that inhibit DNA repair following RT, commonly known as radiosensitizers. The development of radiosensitizing agents against RC is in high demand for the improvement of therapeutic outcomes. Radiosensitizers typically have less of an impact on healthy tissues however, the clinical success of these radiosensitizers is limited. The causes of failing radiosensitizers in clinical trials include difficulty in targeted delivery, lack of optimum absorption in the tumor cells, and targeting a single biological process to achieve the goal. Radiotherapeutic concepts are crucial for the primary management of RC and are a helpful tool in the management of recurrent/metastatic RC.

Therefore, developing a radiosensitizer that can be delivered specifically to tumor cells, formulated using nanotechnology to enhance the bioavailability, and targeted to disrupt multiple biological processes of tumor cells are crucially important. In this regard, nanoparticle-based radio-sensitizers have shown significant progress because of their higher bioavailability, limited cytotoxicity and easy functionalization. A better design of radiosensitizer can be achieved with the help of molecular structure analysis, molecular cloning, and bioinformatics analysis. Finally, the use of artificial intelligence and machine learning in drug discovery for clinical trials may direct the creation of novel radiosensitizers or may improve the efficacy of currently available radiosensitizers.

## Abbreviations

APP: apopicropodophyllin
BEZ235: dactolisib
CAFs: cancer-associated fibroblasts
CEA: carcinoembryonic antigen
CM: conditioned medium
COX-2: cyclooxygenase-2
CSC: cancer stem cell
DDRs: DNA damage responses
DNA-Pk: DNA-dependent protein kinase
DOX: doxorubicin
DSBs: double-strand breaks
EGF: epidermal growth factor
EMT: epithelial-to-mesenchymal transition
EPR: enhanced permeability and retention
FGE$: fibroblast growth factor 4
FOXO: forkhead box O
FTD: trifluridine
H_2_O_2_: hydrogen peroxide
HIF-1: hypoxia Inducing Factor-1
HIF1: hypoxia-inducible factor 1
IGF2: insulin-like growth factor 2
IR: ionizing rRadiation
MCM: minichromosome maintenance
MDT: multidisciplinary treatment
MnSOD: manganese superoxide dismutase
NFk-B: nuclear factor B
NHEJ: non-homologous end-joining
PDGF: platelet-derived growth factors
pGAgNPs: PEGylated GQD-decorated silver nano prisms
PGE2: prostaglandin E2
PHGDH: phosphoglycerate dehydrogenase
PL: piperlongumine
RC: rectal cancer
RNS: reactive nitrogen species
RT: radiotherapy
Ru: ruthenium
SSP: serine synthesis route
TAMs: tumortumour-associated macrophages
TGF- β: transforming growth factor- β
TPI: tipiracil hydrochloride
VEDF: vascular endothelial growth factor

## Novelty in the manuscript

1. The manuscript initially describes the detailed mechanism behind the radiotherapy effects in cancer cells leading to radioresistance.
2. It also describes the mechanisms of radioresistant pathways associated with rectal cancer.
3. Further, the mechanisms behind the radiosensitivity of tumor cells and their microenvironment have been discussed.
4. At the end, it summarizes the therapeutic mechanisms of various radiosensitizers involved in enhancing the radiosensitivity of rectal cancer cells along with radiotherapy.
